# *Calculus Bovis Sativus* Improves Bile Acid Homeostasis via Farnesoid X Receptor-Mediated Signaling in Rats With Estrogen-Induced Cholestasis

**DOI:** 10.3389/fphar.2019.00048

**Published:** 2019-02-01

**Authors:** Dong Xiang, Jinyu Yang, Yanan Liu, Wenxi He, Si Zhang, Xiping Li, Chengliang Zhang, Dong Liu

**Affiliations:** Department of Pharmacy, Tongji Hospital Affiliated, Tongji Medical College, Huazhong University of Science and Technology, Wuhan, China

**Keywords:** *Calculus Bovis Sativus*, cholestasis, FXR, 17α-ethinylestradiol, bile acid

## Abstract

Cholestatic diseases are characterized by toxic bile acid (BA) accumulation, and abnormal BA composition, which subsequently lead to liver injury. Biochemical synthetic *Calculus Bovis Sativus* (CBS) is derived from natural *Calculus Bovis*, a traditional Chinese medicine, which has been used to treat hepatic diseases for thousands of years. Although it has been shown that CBS administration to 17α-ethinylestradiol (EE)-induced cholestatic rats improves bile flow and liver injury, the involved underlying mechanism is largely unknown. In this study, we showed that CBS administration to EE-induced cholestatic rats significantly decreased serum and hepatic BA levels and reversed hepatic BA composition. DNA microarray analysis suggested that the critical pathways enriched by CBS treatment were bile secretion and primary BA synthesis. These findings led us to focus on the effects of CBS on regulating BA homeostasis, including BA transport, synthesis and metabolism. CBS enhanced hepatic BA secretion by inducing efflux transporter expression and inhibiting uptake transporter expression. Moreover, CBS reduced BA synthesis by repressing the expression of BA synthetic enzymes, CYP7A1 and CYP8B1, and increased BA metabolism by inducing the expression of metabolic enzymes, CYP3A2, CYP2B10, and SULT2A1. Mechanistic studies indicated that CBS increased protein expression and nuclear translocation of hepatic and intestinal farnesoid X receptor (FXR) to regulate the expression of these transporters and enzymes. We further demonstrated that beneficial effects of CBS administration on EE-induced cholestatic rats were significantly blocked by guggulsterone, a FXR antagonist. Therefore, CBS improved BA homeostasis through FXR-mediated signaling in estrogen-induced cholestatic rats. Together, these findings suggested that CBS might be a novel and potentially effective drug for the treatment of cholestasis.

## Introduction

Cholestatic liver diseases, including estrogen-induced cholestasis, primary biliary cholangitis (PBC), and primary sclerosing cholangitis (PSC), result from intrahepatic accumulation of toxic bile acids (BAs) that cause liver injury and ultimately lead to fibrosis and cirrhosis (Trauner et al., 2017). When treatment is delayed, many patients would require a liver transplantation at the end-stage of cholestatic diseases (Wang et al., 2017). Estrogens and their metabolites are known to cause intrahepatic cholestasis in susceptible women during pregnancy as well as administration of oral contraceptives and postmenopausal hormone replacement therapy (Li et al., 2017). Currently, only few therapies to effectively treat cholestatic diseases are available, and the curative effect is limited (Jansen et al., 2017). Thus, developing novel therapeutic strategies for treating and managing cholestatic liver diseases is of utmost importance.

Considering clinical implications, experimental cholestasis induced by 17α-ethynylestradiol (EE) administration in rodents has been widely used to investigate the underlying molecular/cellular mechanisms involved in estrogen-induced cholestasis (Zhang et al., 2018). In experimental animals, treatment with EE decreased bile flow and BA synthesis, thereby leading to accumulation of high levels of BAs and an abnormal BA composition in the liver (Fischer et al., 1996). BA homeostasis is tightly controlled by hepatic nuclear receptors, including the farnesoid X receptor (FXR, NR1H4), pregnane X receptor (PXR, NR1I2), vitamin D receptor (VDR, NR1I1), constitutive androstane receptor (CAR, NR1I3), liver X receptor (LXR, NR1H3), and the peroxisome proliferator-activated receptor α (PPARα, PPARA) (Zollner and Trauner, 2009). Importantly, FXR is one of the main mediators in the liver and intestine and regulates BA transporters and enzymes to maintain BA homeostasis Li and Chiang, 2013. Ligand-activated FXR promoted BA secretion through inducing the expression of efflux transporters, including bile salt export pump (BSEP, ABCB11) and multidrug resistance-associated protein 2 (MRP2, ABCC2), and reduced BA reabsorption via decreasing the expression of uptake transporters, such as Na^+^-dependent taurocholate cotransport peptide (NTCP), and sodium-independent organic anion transporters (OATPs) (Halilbasic et al., 2013). In addition, FXR inhibited cholesterol 7α-hydroxylase (CYP7A1) and microsomal sterol 12α-hydroxylase (CYP8B1) to reduce BA synthesis and induce CYP3A2 (a homolog of human CYP3A4) and sulfotransferase family 1E member 1 (SULT1E1) to promote BA detoxification (Chiang, 2013). In previous studies, it has been demonstrated that targeting FXR is promising for treating cholestatic liver diseases (Beuers et al., 2015).

*Calculus Bovis* is a traditional Chinese medicine, which has been widely used in Oriental and Southeast Asian countries, especially China, to relieve fever, alleviate inflammation, maintain sedation, and recover gallbladder function (Chen, 1987). Due to the extremely scarce resources (gallstones of *Bos Taurus domesticus Gmelin*), and high prices, countless studies have strived to substitute natural *Calculus Bovis*. According to the formation principle of gallstones *in vivo* and the methods of biochemical synthesis, *Calculus Bovis Sativus* (CBS) is cultivated from bovine bile *in vitro* by modern bioengineering techniques. CBS is an ideal substitute, which is identical in terms of properties and components and has been included in the Pharmacopoeia of the People’s Republic of China since 2005 Edition (Feng et al., 2015). According to pharmacopeia, CBS has a definite chemical profile, containing cholic acid, deoxycholic acid, and bilirubin as its principal bioactive components. As reported, CBS has been successfully used to treat many severe liver diseases, such as hepatic cancer, hepatitis B, and non-alcoholic fatty liver disease in both humans and experimental animals (Liu et al., 2010; Bai and Zhao, 2011; Cheng et al., 2014). In our previous study, we showed that CBS exerted beneficial effects on EE-induced cholestatic rats, at least in part by improving functions of MRP2, breast cancer resistance protein (BCRP), and P-glycoprotein (P-gp) (Liu et al., 2014).

In the present study, we aimed to fully understand the hepatoprotective effects of CBS on EE-induced cholestasis and investigated the underlying mechanisms involved. We utilized DNA microarray to screen the critical pathways involved in CBS treatment and studied the effects of CBS on hepatic BA composition as well as regulatory functions on BA synthesis, metabolism, and transport. The findings of our study suggested that FXR-mediated signaling plays a critical role in the beneficial effects of CBS on EE-induced cholestasis.

## Materials and Methods

### Chemicals and Reagents

*Calculus Bovis Sativus* was provided by Wuhan Jianmin Dapeng Pharmaceutical Co., Ltd. (Lot: 2016-05-16, Wuhan, China), and the same preparation and lot number was used in our previous study (He et al., 2017). We also successfully established a liquid chromatography-tandem mass spectrometry (LC-MS/MS) approach to determine the main components in CBS, and subsequently established a quality control method to ensure the stability, uniformity, and quality of CBS (Feng et al., 2015; He et al., 2017). EE was purchased from Sigma-Aldrich (St. Louis, MO, United States, purity ≥98%). Guggulsterones (GS) was purchased from Dibo biochemical Co., Ltd. (Shanghai, China). Standards for BAs for cholic acid (CA), deoxycholic acid (DCA), chenodeoxycholic acid (CDCA), β-muricholic acid (β-MCA), taurocholic acid (TCA), taurodeoxycholic acid (TDCA), β-tauromuricholic acid (Tβ-MCA), and tauroursodeoxycholic acid (TUDCA) were obtained from Steraloids (Newport, RI, United States). The internal standard (IS) was from Isoreag (Shanghai, China). Antibodies directed against NTCP, FXR, CYP7A1, LXR, VDR, CAR, PXR, PPARα, CK19, Lamin B, and β-actin were purchased from Absin Biochemical Company (Shanghai, China). An antibody directed against BSEP was obtained from Santa Cruz Biotechnology (Santa Cruz, CA, United States) and an antibody directed against MRP2 was obtained from Abcam (Cambridge, United Kingdom). All other chemical agents used were from analytical grade or HPLC grade.

### Animals and Treatments

The present study was conducted in strict accordance with the National Institutes of Health guide for the care and use of laboratory animals (Muhler et al., 2011). The animal protocol was approved by the Ethical Committee on Animal Experimentation of the Tongji hospital (Tongji, Wuhan, China). Male Sprague-Dawley rats, 8 weeks of age and weighing 220 ± 20 g were obtained from the Center of Experimental Animal of Hubei Province (Wuhan, China). All animals were housed under standard laboratory conditions at a temperature of 25 ± 2°C and a 12-h light/dark cycle. To induce cholestatic rats, EE (5 mg/kg) was subcutaneously injected for five consecutive days. Non-cholestatic control rats received the EE vehicle (propylene glycol). CBS (150 mg/kg) or vehicle (0.5% sodium carboxymethyl cellulose) was administered to rats by oral gavage once per day for five consecutive days with co-administration of EE or EE vehicle. GS, an FXR antagonist, was dissolved as previous described (Meng et al., 2015), and rats were injected intraperitoneally with 10 mg/kg of GS 4 h prior to CBS or vehicle treatment. Body weight was recorded daily. Animals were fasted overnight and sacrificed randomly between 8:00 and 11:00 am. Blood, bile, liver, and ileum samples were collected for further analyses.

### Serum Biochemistry Assay and Histology

Serum levels of alanine aminotransferase (ALT), aspartate aminotransferase (AST), alkaline phosphatase (ALP) as well as serum and hepatic levels of total bile acid (TBA) were determined using commercial kits (JianCheng, Nanjing, China) according to the manufacturer’s instructions. After rats were sacrificed, livers were collected, fixed in 4% formaldehyde, embedded in paraffin, sectioned at 5 μm, and stained with hematoxylin and eosin (H&E). Images were taken by EVOS FL Auto microscope (Life Technologies, Carlsbad, CA, United States) and Olympus microscope (Olympus, Tokyo, Japan).

### DNA Microarray Analysis

Total RNA of three randomly chosen livers from non-cholestatic, EE, and EE+CBS rats was isolated by a Takara RNAiso Plus kit and purified using an RNeasy Mini Kit (Qiagen, GmBH, Darmstadt, Germany) following the manufacturer’s instructions. Total RNA was amplified and labeled by an Agilent Quick Amp Labeling Kit. Each slide was hybridized with Agilent Whole Rat Genome Oligo Microarray (4 × 44K). Data were collected by Agilent Feature Extraction software 10.7 (Agilent Technologies, Santa Clara, CA, United States) and then filtered for significant detection (Student’s *t*-test, *p* < 0.05, and fold change >1.2 or <0.8). Technical support was provided by Biotechnology Corporation (Shanghai, China) for determining the whole gene expression profile. Gene ontology (GO) and Kyoto encyclopedia of genes and genomes (KEGG) analyses of differentially expressed genes were performed by using the common public online database DAVID Bioinformatics Resources 6.8 Tools^1^.

### Analysis of Bile Acids in the Liver by Liquid Chromatography-Tandem Mass Spectrometry

In brief, approximately 100 mg of liver tissue was homogenized in 4 volumes of methanol-water (50:50 v/v). A total of 150 μL of liver homogenate was added to 20 μL IS (5 μmol/L d4-GCDCA) and 1 mL of ice-cold alkaline acetonitrile (containing 5% NH_3_⋅H_2_O v/v), then samples were vortexed and shaken for 60 min, and centrifuged at 12,000 ×*g* for 10 min at 4°C. Subsequently, supernatants were evaporated and reconstituted in 100 μL methanol. A high-performance liquid chromatography (HPLC; LC-20AD, Shimadzu, Japan) system coupled to an Applied Biosystems 4000 Q trap (AB Sciex, CA, United States) mass spectrometer with electrospray ionization source (ESI) was used. Chromatographic separation of BAs was performed using a C18 column (3.5 μm, 2.1 mm × 150 mm, Symmetry^®^Waters, MA, United States). The mobile phase consisted of A (water with 10 mM ammonium acetate and 0.1% formic acid) and B (methanol with 10 mM ammonium acetate and 0.1% formic acid). A gradient elution was used, starting with 60% B for the first 2 min, then linearly increased to 90% B in 38 min, and kept constant for 5 min. All BAs were detected in the negative ionization mode with the following mass spectrometer source settings: ion spray voltage = -4500 V; ionsource heater = 300°C; source gas 1 = 40 psi; source gas 2 = 40 psi, and curtain gas = 20 psi. Data were collected and analyzed using Analyst 1.6.1 software (AB Sciex, CA, United States).

### Quantitative Real-Time PCR Assay

Total RNA from liver and intestine was extracted using TRIzol Reagent (Invitrogen, CA, United States) according to the manufacturer’s instructions. Total RNA was reverse transcribed into cDNA using PrimeScript™ RT Master Mix (Takara, Dalian, China), and then obtained cDNA was used as a template for real-time PCR amplification, performed by using SYBR Green (Takara, Dalian, China) and forward/reverse primer pairs for the tested genes. Independent reactions were performed in triplicate using an ABI StepOne Plus system (Applied Biosystems, CA, United States). Threshold cycle values were normalized to β-actin. Both forward and reverse primers used are presented in Supplementary Table 1.

### Western Blot Analysis

Western blot analysis of membrane and total protein samples was performed as described in our previous study (Liu et al., 2014). In brief, nuclear protein samples from hepatic and intestinal tissues were extracted following standard protocols (Beyotime Institute of Biotechnology, Shanghai, China). Proteins were subjected to SDS–PAGE, then transferred to PVDF membranes. After blocking for 1 h with 5% non-fat milk in TBST, membranes were incubated overnight at 4°C with primary antibodies directed against MRP2, BSEP, NTCP, CYP7A1, FXR, LXR, VDR, CAR, PXR, PPARα, Lamin B, and β-actin. Next, membranes were incubated with horseradish peroxidase (HRP)-conjugated antibodies for 1 h at room temperature at a 1:2500 dilution. Protein expression was evaluated by an enhanced chemiluminescence (ECL) approach and membranes were imaged with a BOX Chemi XRQ imaging system (SynGene, Cambridge, United Kingdom).

### Immunohistochemistry

Immunohistochemical analysis for CK19, FXR, and CYP7A1 was performed as previously described (Liu et al., 2014). In brief, hepatic or intestinal tissues sections were incubated with antibodies directed against CK19, FXR, or CYP7A1 for 5 h at 37°C, then treated with corresponding secondary antibodies. Images were taken using an Olympus microscope (Tokyo, Japan), and analyzed by Image-Pro Plus 6.0 software (Media Cybernetics, Silver Spring, MD, United States). Positive staining was quantified by counting three different fields per section (200×).

### Statistical Analysis

Data are expressed as the mean ± SE. Significance was determined by one-way analysis of variance (ANOVA) followed by Tukey’s test using GraphPad Prism 7 software. *P* < 0.05 was considered statistically significant.

## Results

### CBS Alleviates Liver Injury and Reduces Serum Bile Acid Levels

**FIGURE 1:**
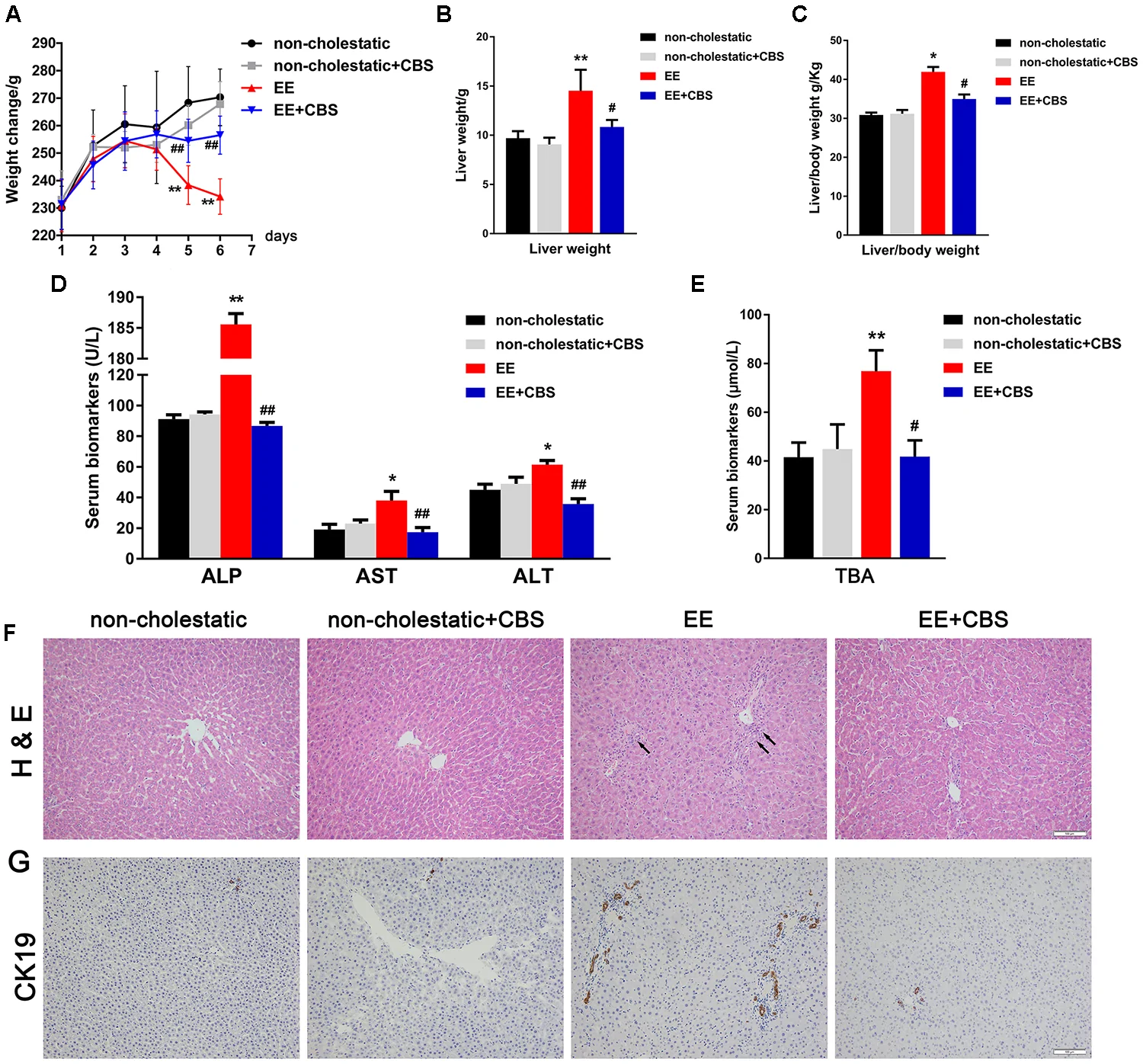
CBS alleviates liver injury and reduces serum bile acid levels in rats with 17α-ethinylestradiol-induced cholestasis. **(A)** Changes in body weight in each group during the six experimental days. Determination of **(B)** liver weight, **(C)** liver/body weight, and **(D)** serum alanine aminotransferase (ALT), aspartate aminotransferase (AST), alkaline phosphatase (ALP), and **(E)** total bile acid (BA) levels. **(F)** Hematoxylin and eosin (H&E) staining was used to investigate hepatic structural changes under non-cholestatic and cholestatic conditions in rats. **(G)** Immunohistochemistry of CK19 was performed to evaluate proliferation of the bile duct. Data are presented as the mean ± SD (*n* = 6). Significant differences compared with the non-cholestatic group, ^∗^*p* < 0.05; ^∗∗^*p* < 0.01; compared with the 17α-ethinylestradiol (EE) group, ^#^*p* < 0.05; ^##^*p* < 0.01.

In our preliminary study, we found the best hepatoprotective effects of CBS in the high dose group. Therefore, in the present study, 150 mg/kg of CBS was chosen. At the first 4 days, the body weight of the animals in the four groups was not significantly changed. However, at days 5 and 6, the body weight of EE cholestatic rats was markedly reduced when compared to that of non-cholestatic rats. In contrast, the body weight of cholestatic rats treated with CBS was slightly reduced but not significantly different from the body weight of non-cholestatic rats (Figure 1A). This suggested that CBS treatment improved the overall condition of cholestatic animals. This beneficial effect was also noted in the liver weight and liver/body weight: the weight or relative weight of livers of rats in the EE+CBS group was markedly lower when compared to that of rats in the EE group (Figures 1B,C).

As previously reported (Liu et al., 2014; Meng et al., 2015), serum ALT, AST, and ALP levels were significantly higher in EE cholestatic rats when compared to that of non-cholestatic rats. These biochemical indicators of hepatotoxicity were reduced by CBS treatment (Figure 1D). Serum TBA levels were markedly higher in EE cholestatic rats, whereas CBS administration resulted in a striking reduction in serum TBA levels in cholestatic rats (Figure 1E).

Histological assessments of the liver further indicated EE-induced hepatotoxicity. EE groups associated with significant increases in inflammatory cell infiltration, edema, bile duct proliferation, and severe hepatic necrosis (Figure 1F). Immunohistochemistry of CK19 further confirmed the bile duct proliferation in EE groups. These pathological changes were markedly reduced by CBS treatment (Figure 1G and Supplementary Figure 1). CBS administration in non-cholestatic rats did not cause significant changes in body weight, liver weight, liver/body weight, serum biomarkers, or liver histology.

### CBS Improves Intrahepatic Bile Acid Accumulation and Composition

**FIGURE 2:**
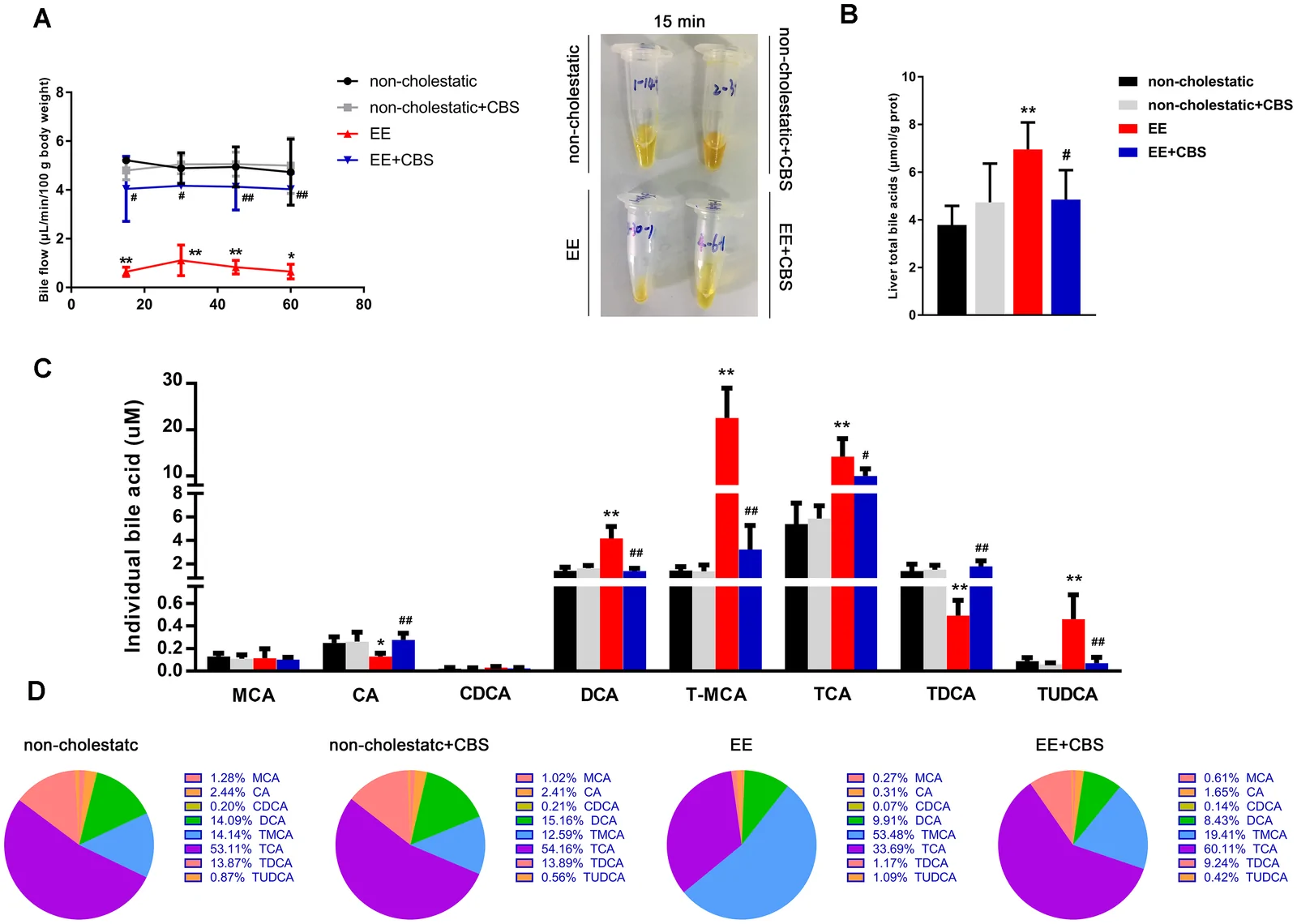
CBS improves intrahepatic bile acid accumulation and composition. **(A)** Bile was collected for four 15-min periods over 60 min under basal, non-stimulated conditions. The bile flow rate was calculated by gravimetry, assuming the density of the bile of 1.0 g/mL. Representative collection tubes are shown here. **(B)** Liver total bile acids (BAs). **(C)** Hepatic individual BAs were assessed by liquid chromatography-tandem mass spectrometry (LC-MS/MS). **(D)** Analysis of BA composition. Data are presented as the mean ± SD (*n* = 6). Significant differences when compared with the non-cholestatic group, ^∗^*p* < 0.05; ^∗∗^*p* < 0.01; compared with the 17α-ethinylestradiol (EE) group, ^#^*p* < 0.05; ^##^*p* < 0.01. Abbreviations: MCA, muricholic acid; CA, cholic acid; CDCA, chenodeoxycholic acid; DCA, deoxycholic acid; TMCA, tauromuricholic acid; TCA, taurocholic acid; TDCA, taurodeoxycholic acid; TUDCA, tauroursodeoxycholic acid.

Figure 2 shows the basal bile flow, liver total BAs as well as hepatic BA composition in EE cholestatic and non-cholestatic rats treated with CBS or vehicle. As expected, remarkable bile flow obstruction (approximately down to 16.3%) and hepatic BA accumulation (up to 183.7%) were observed in EE-induced cholestasis when compared with non-cholestatic rats. However, CBS administration significantly increased bile flow 5.0-fold and decreased liver total BAs 1.4-fold in EE cholestatic rats (Figures 2A,B). Individual BA levels and BA composition was performed in liver tissues using an LC-MS/MS method. Representative chromatograms are shown in Supplementary Figure 2 and the method details are provided in Supplementary Tables 2, 3. Together, these results suggested that EE-induced rats increased hepatic DCA, TMCA, TCA, and TUDCA levels, and decreased CA and TDCA levels, as well as impaired BA composition (Figures 2C,D). However, CBS administration reversed individual BA levels (Figure 2C) and nearly shifted the abnormal BA composition to non-cholestatic level (Figure 2D).

### Gene Expression Profile of CBS on EE-Induced Cholestasis

**FIGURE 3:**
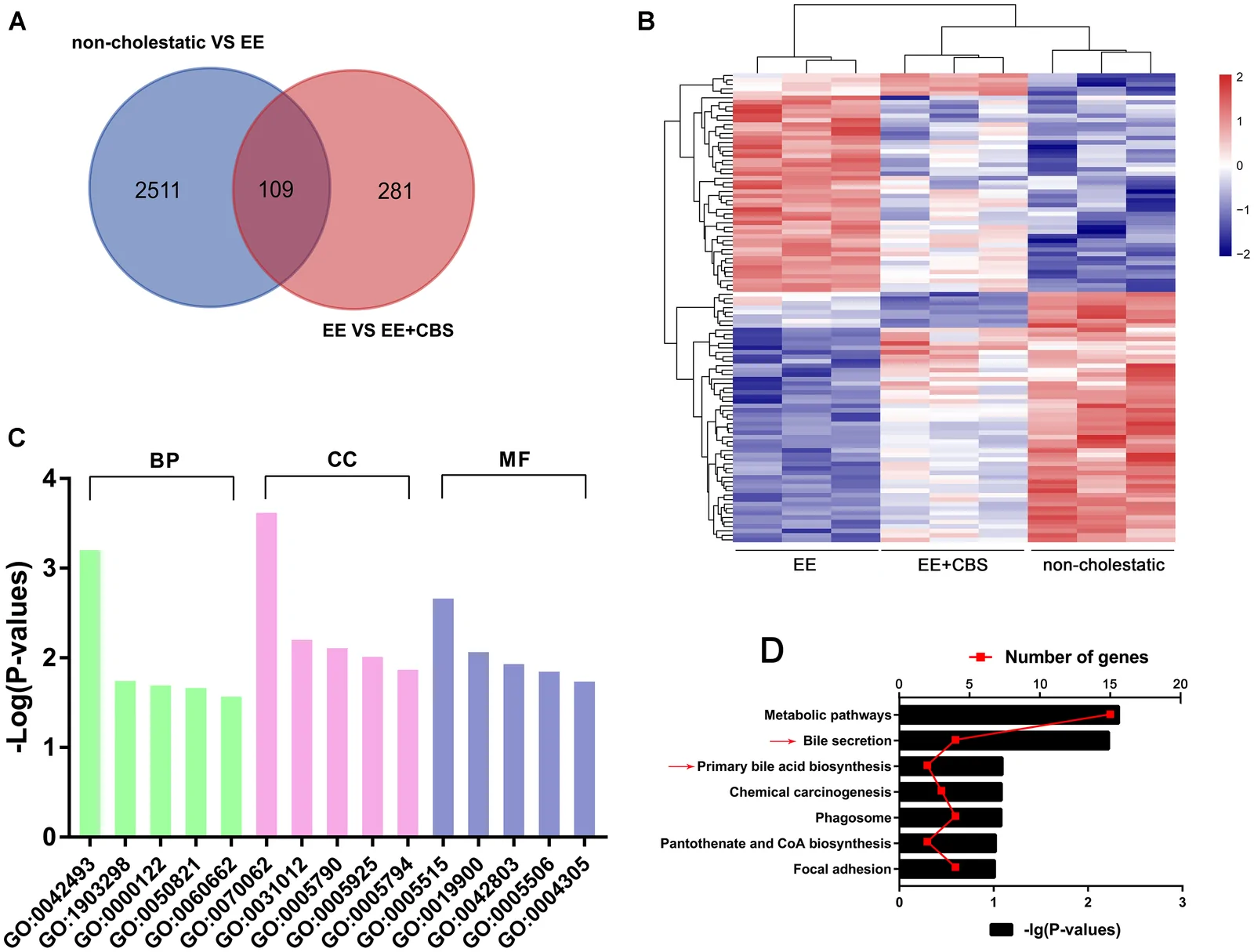
DNA microarray analysis of non-cholestatic, 17α-ethinylestradiol (EE) and EE+CBS rat livers. **(A)** Venn diagram analysis of differentially expressed genes of non-cholestatic, EE and EE+CBS groups. **(B)** Heat map of differentially expressed genes in non-cholestatic, EE and EE+CBS groups **(C)** gene ontology (GO) analysis of differentially expressed genes **(D)** Kyoto encyclopedia of genes and genomes (KEGG) pathway analysis of differentially expressed genes. Arrows point to the pathways related to bile acid (BA) homeostasis; (*n* = 3). Abbreviations: BP, biological process; CC, cellular component; MF, molecular function. Interpretation: GO:0042493, response to drug; GO:1903298, negative regulation of hypoxia-induced intrinsic apoptotic signaling pathway; GO:0000122, negative regulation of transcription from RNA polymerase II promoter; GO:0050821, protein stabilization; GO:0060662, salivary gland cavitation; GO:0070062, extracellular exosome; GO:0031012, extracellular matrix; GO:0005790, smooth endoplasmic reticulum; GO:0005925, focal adhesion; GO:0005794, Golgi apparatus; GO:0005515, protein binding; GO:0019900, kinase binding; GO:0042803, protein homodimerization activity; GO:0005506, iron ion binding; and GO:0004305, ethanolamine kinase activity.

To systematically investigate the molecular mechanisms underlying the hepatoprotective effects of CBS on EE-induced cholestasis, we first performed DNA microarray analysis. The results of the significant difference (*p* < 0.05 and fold change >1.2) gene showed that: compared with non-cholestatic rats, there were 2511 differentially expressed genes in EE cholestatic rats. In addition, compared with EE cholestatic rats, there were 281 differentially expressed genes in CBS-treated cholestatic rats. When comparing all three groups, a total of 109 genes overlapped (Figure 3A). The heatmap of 109 differentially expressed genes is presented in Figure 3B. GO analysis suggested that the main biological process involved a response to drugs (GO: 0042493), the molecular function primary involved protein binding (GO: 0070062), and the main cellular component was associated with extracellular exosome (GO: 0005515) (Figure 3C and Supplementary Table 4). The KEGG pathways were mainly enriched in metabolic pathways, bile secretion, primary BA biosynthesis, and chemical carcinogenesis (Figure 3D and Supplementary Table 5). In conclusion, CBS improved intrahepatic BA accumulation involved in regulating multiple pathways.

### CBS Promotes Bile Acid Efflux and Reduces Bile Acid Influx

Because CBS improved hepatic BA accumulation, and the gene expression profile of CBS treatment was enriched in bile secretion and primary BA biosynthesis (Figure 3D), we hypothesized that CBS alleviating EE-induced cholestasis might be involved in the modulation of BA homeostasis, including BA transport, metabolism, and synthesis. Therefore, we first evaluated changes in expression of various BA transporters after CBS administration.

**FIGURE 4:**
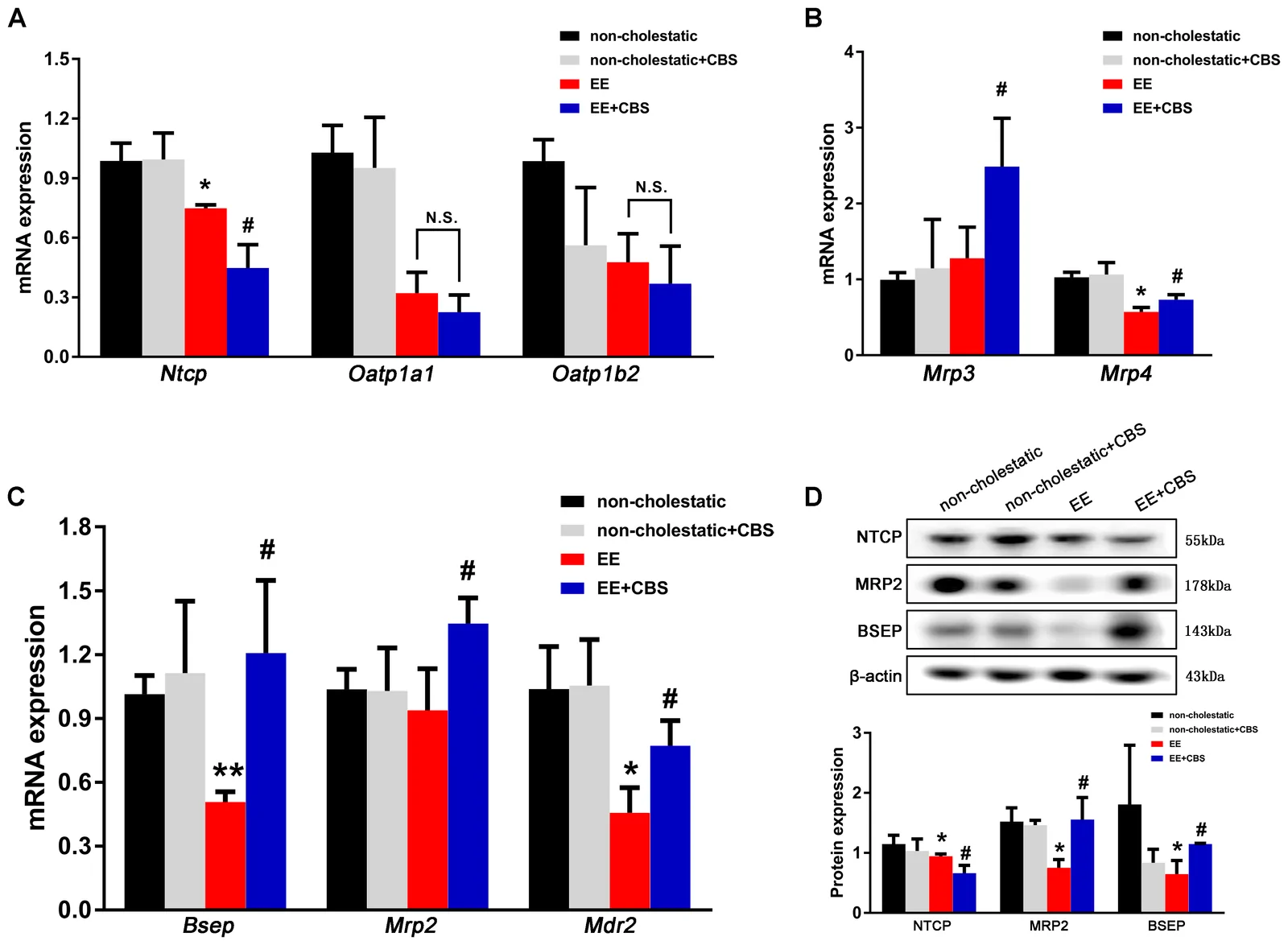
CBS promotes bile acid efflux and reduces bile acid influx in cholestatic rats. mRNA expression of **(A)** basolateral uptake transporters, *Ntcp, Oatp1a1*, and *Oatp1b2*, as well as **(B)** basolateral efflux transporters, *Mrp3* and *Mrp4*, and **(C)** canalicular efflux transporters, *Bsep, Mrp2*, and *Mdr2*, was determined by real-time PCR and normalized to β-actin. **(D)** Protein levels of NTCP, BSEP, and MRP2 were determined by Western blot analysis and normalized with β-actin. Data are presented as the mean ± SD (*n* = 6). Significant differences compared to the non-cholestatic group, ^∗^*p* < 0.05; ^∗∗^*p* < 0.01; compared with the 17α-ethinylestradiol (EE) group, ^#^*p* < 0.05; ^##^ *p* < 0.01. N.S., no significance.

The basolateral uptake transporter Ntcp showed a significant decrease in CBS administration when compared to EE cholestatic rats, whereas Oatp1a1 and Oatp1b2 were unaffected (Figure 4A). In addition, CBS administration in EE-treated rats significantly induced mRNA expression of *Mrp3* and *Mrp4* in the liver, which predominantly secreted BAs into the circulation (Figure 4B). However, apical transporters such as *Bsep, Mrp2*, and *Mdr2* decreased in rats after EE treatment but increased after CBS administration when compared to EE cholestatic rats (Figure 4C). Western blot results for the main efflux and influx transporters corresponded with real-time PCR findings. The protein expression of BSEP, MRP2, and NTCP was decreased in EE cholestatic rats, whereas NTCP levels further reduced, and MRP2 and BSEP levels markedly increased with CBS treatment (Figure 4D). Thus, CBS protected the liver from toxic BA accumulation involved in decreasing BA uptake from the portal circulation and increased the excretion of BA from hepatocytes.

### CBS Reduces Bile Acid Synthesis and Promotes Bile Acid Metabolism

**FIGURE 5:**
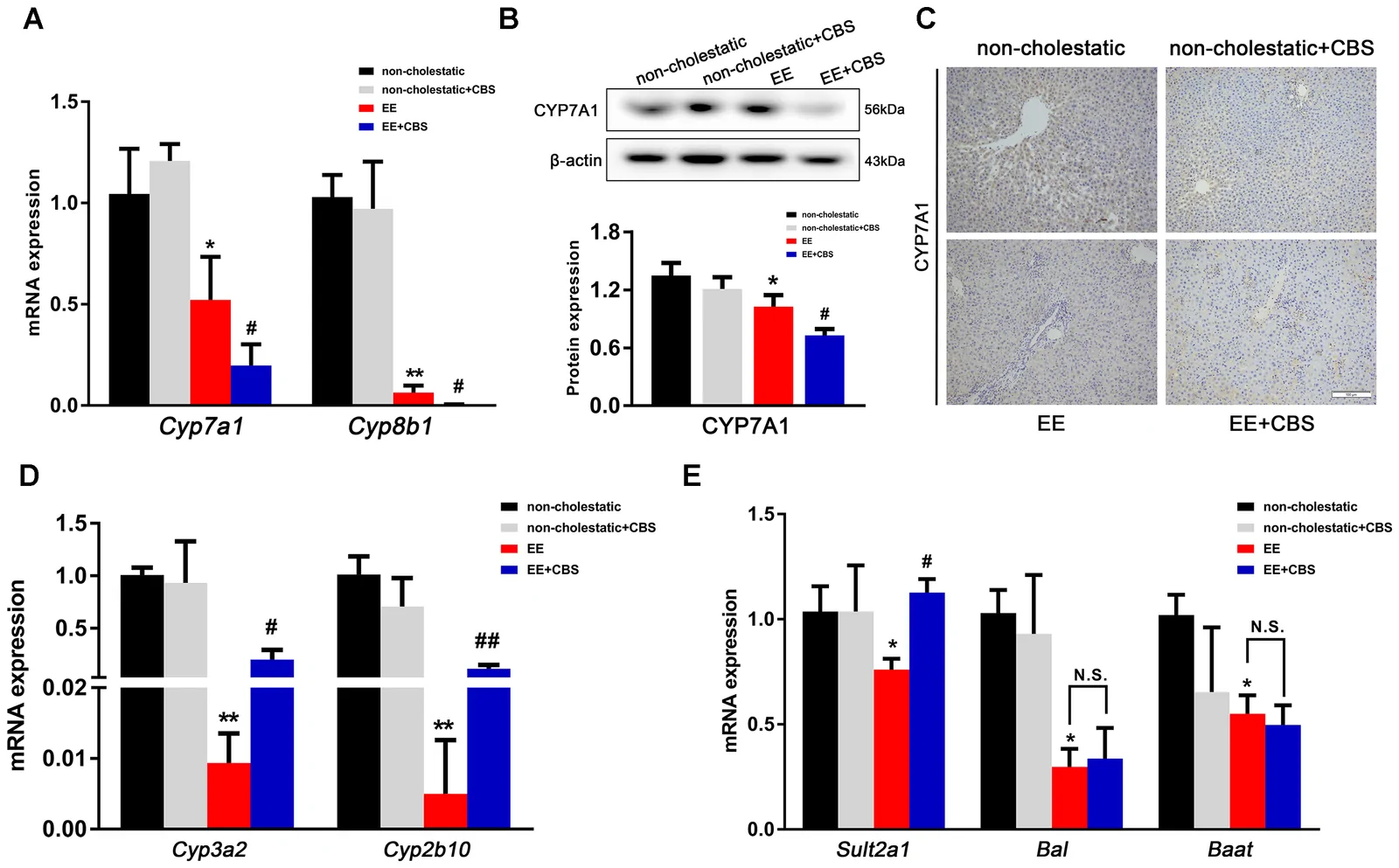
CBS reduces bile acid synthesis and promotes bile acid metabolism in cholestatic rats. **(A)** mRNA expression of bile acid (BA) synthetic enzymes, *Cyp7a1* and *Cyp8b1*, was determined by real-time PCR and normalized to β-actin. **(B)** Protein expression of hepatic CYP7A1 was determined by Western blot analysis and normalized to β-actin. **(C)** Representative images of immunohistochemical staining of hepatic CYP7A1. mRNA expression of **(D)** *Cyp3a2, Cyp2b10* and **(E)** *Sult2a1, Bal*, and *Baat* was evaluated by real-time PCR and normalized to β-actin. Data are presented as the mean ± SD (*n* = 6). Significant differences compared with the non-cholestatic group, ^∗^*p* < 0.05; ^∗∗^*p* < 0.01; compared with the 17α-ethinylestradiol (EE) group, ^#^*p* < 0.05; ^##^*p* < 0.01. N.S., no significance.

Next, we investigated BA synthesis and metabolism. As shown in Figure 5A, mRNA levels of *Cyp7a1* and *Cyp8b1*, two enzymes involved in the classic pathway of BA synthesis, were significantly reduced by 50 and 90%, respectively, in EE-induced cholestatic rats when compared to non-cholestatic rats. In addition, CBS administration further inhibited the transcription of *Cyp7a1*, and completely abolished *Cyp8b1* expression. Western blot analysis showed that CYP7A1 was modestly but markedly decreased in CBS-treated livers when compared to the EE group (Figure 5B). Moreover, immunohistochemical staining of CYP7A1 further confirmed these results (Figure 5C and Supplementary Figure 3).

It has been shown that phase I (CYP3A2 and CYP2B10) and phase II (SULT2A1, BAL and BAAT) metabolic enzymes mainly mediate BAs detoxification in the liver (Li and Chiang, 2013). In our study, we showed that expression levels of *Cyp3a2* and *Cyp2b10* mRNA were dramatically decreased in EE-induced cholestatic rats, which were mildly, but markedly increased by CBS treatment (Figure 5D). EE decreased mRNA expression of *Sult2a1, Bal*, and *Baat*, whereas CBS administration significantly enhanced *Sult2a1* expression level but did not affect *Bal* and *Baat* (Figure 5E). Taken together, CBS not only improved BA transport but CBS also decreased BA synthesis and increased BA phase I and phase II metabolism in the liver, with the net result being decreased accumulation of toxic BAs.

### CBS Activates Protein Expression and Nuclear Translocation of FXR in the Liver and Intestine

**FIGURE 6:**
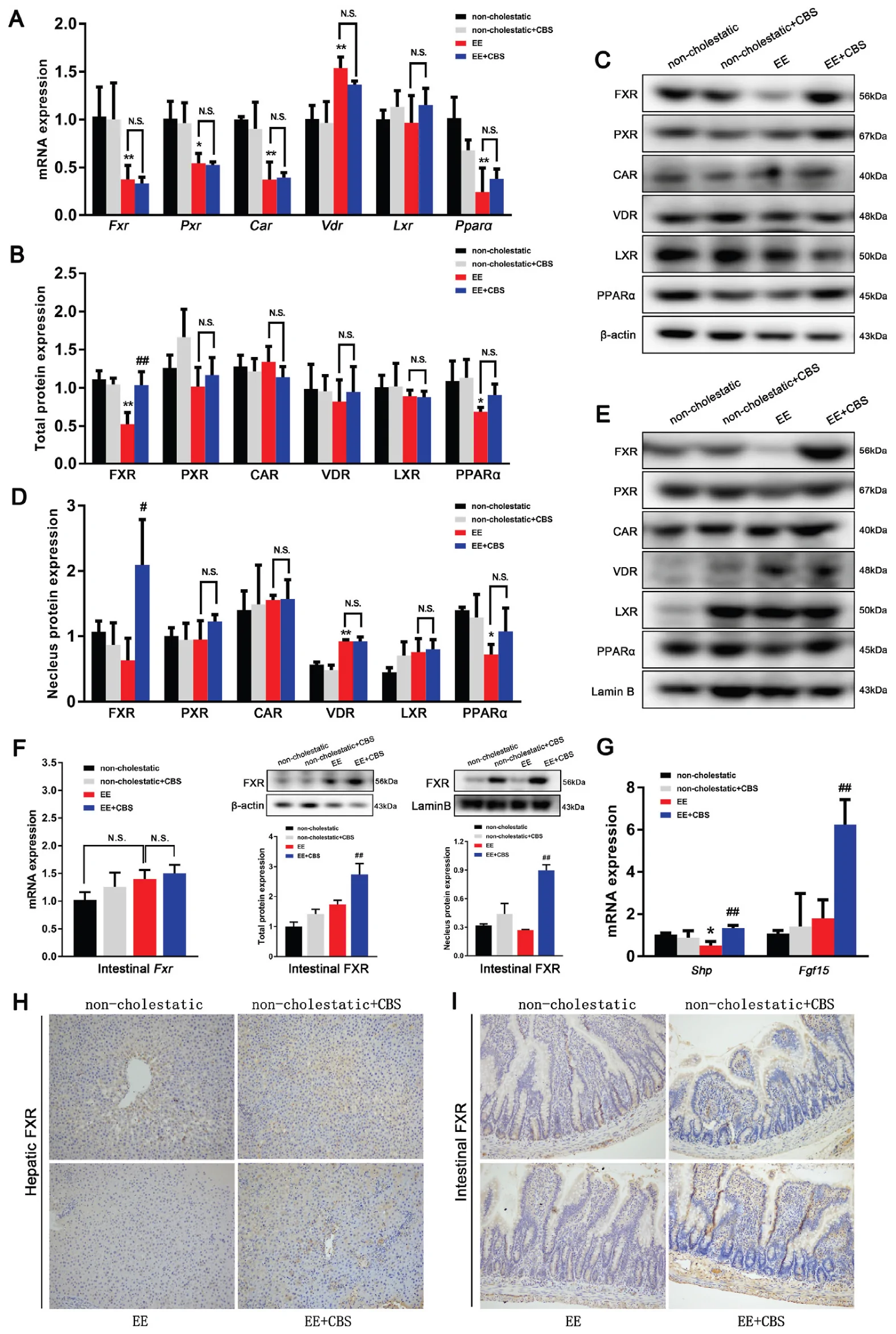
CBS activates the protein expression and nuclear translocation of FXR in liver and intestine. **(A)** mRNA expression of hepatic nuclear receptors *Fxr, Pxr, Car, Vdr, Lxr*, and *Pparα* was determined by real-time PCR and normalized to β-actin. **(B,C)** Total protein levels and **(D,E)** nuclear protein levels of hepatic FXR, PXR, CAR, VDR, LXR, and PPARα were determined by Western blot analysis and normalized to β-actin and Lamin B. Representative immunoblot images are shown. **(F)** mRNA, total protein and nuclear protein levels of intestinal FXR were determined using real-time PCR and Western blot analysis and normalized to β-actin and Lamin B. **(G)** mRNA levels of *Shp* in liver and *Fgf15* in intestine were evaluated by real-time PCR and normalized to β-actin. Representative images of immunohistochemical staining of **(H)** hepatic FXR and **(I)** intestinal FXR. Data are presented as the mean ± SD (*n* = 6). Significant differences compared with the non-cholestatic group, ^∗^*p* < 0.05; ^∗∗^*p* < 0.01; compared with the 17α-ethinylestradiol (EE) group, ^#^*p* < 0.05; ^##^*p* < 0.01. N.S., no significance.

Numerous studies have demonstrated that liver nuclear receptors including FXR, PXR, CAR, VDR, LXR, and PPARα, play important roles in BA homeostasis through controlling BA synthesis, metabolism, and transport (Li and Chiang, 2013). CBS alleviated EE-induced cholestasis, which was associated with mediating hepatic BA transporters and enzymes, therefore we next examined whether those nuclear receptors were involved in this regulatory progress. Unexpectedly, microarray analysis showed that there was no significant alteration between the EE+CBS group and EE group among these nuclear receptors (Supplementary Figure 4), which was consistent the real-time PCR results (Figure 6A). Interestingly, Western blot analysis showed that FXR was dramatically decreased by 50% in EE cholestatic rats, but was totally reversed to the non-cholestatic level after CBS treatment (Figures 6B,C). Furthermore, CBS administration significantly increased FXR nuclear translocation in cholestatic rats (Figures 6D,E). However, protein levels and nuclear translocation of other nuclear receptors did not change in CBS-treated cholestatic rats when compared with EE cholestatic rats (Figures 6B–E). Moreover, hepatic small heterodimer partner (SHP), a direct target gene of FXR, was markedly increased (Figure 6G). These results may indicate that FXR-mediated signaling, but not PXR, CAR, VDR, LXR, and PPARα, play a critical role in CBS administration in EE-induced cholestatic rats.

In our study, CYP7A1 was severely inhibited in the rat liver. Recent studies have shown that hepatic CYP7A1 is down-regulated by both hepatic FXR-SHP and intestinal FXR-fibroblast growth factor 15 (FGF15)-mediated pathways (Kong et al., 2012). Therefore, we investigated if intestinal FXR participated in CBS treatment in EE cholestatic rats. Consistent with the liver observations, CBS administration did not alter mRNA expression of intestinal *Fxr*, but dramatically increased protein expression and nuclear translocation of intestinal FXR (Figure 6F), and increased mRNA expression of its target gene *Fgf15* (Figure 6G). In addition, immunohistochemical staining showed increased expression of hepatic and intestinal FXR after CBS treatment (Figures 6H,I and Supplementary Figure 5). Thus, CBS administration activated both hepatic and intestinal FXR and increased their nuclear translocation to regulate the expression of BA transporters and enzymes.

### CBS Improved Hepatic Bile Acid Homeostasis Is Abrogated by FXR Antagonist GS

**FIGURE 7:**
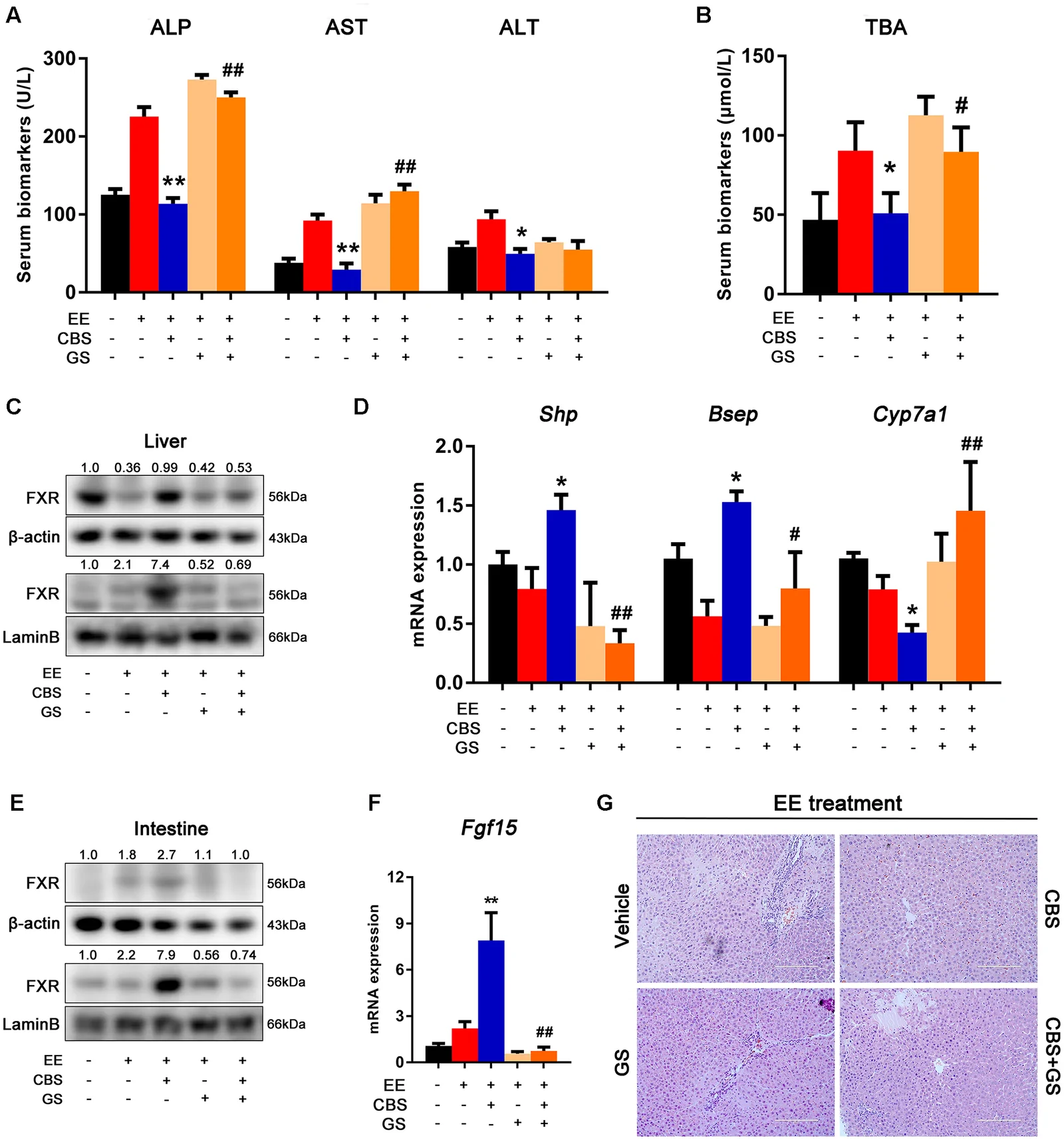
CBS improved hepatic bile acid homeostasis is abrogated by FXR antagonist GS. **(A)** Serum levels of alanine aminotransferase (ALT), aspartate aminotransferase (AST), and alkaline phosphatase (ALP). **(B)** Serum levels of liver total bile acids (TBA). **(C)** Total and nuclear protein levels of hepatic FXR were determined by Western blot analysis and normalized to β-actin and Lamin B. **(D)** mRNA expression of hepatic *Shp, Bsep* and *Cyp7a1* were evaluated by real-time PCR and normalized to β-actin. **(E)** Total and nuclear protein levels of intestinal FXR were determined by Western blot analysis and normalized to β-actin and Lamin B. **(F)** mRNA expression of intestinal *Fgf15* was determined by real-time PCR and normalized to β-actin. **(G)** Representative images of hematoxylin and eosin straining. Data are presented as the mean ± SD (*n* = 6). Significant differences compared with the non-cholestatic group, ^∗^*p* < 0.05; ^∗∗^*p* < 0.01; compared with the 17α-ethinylestradiol (EE) group, ^#^*p* < 0.05; ^##^*p* < 0.01.

To further confirm that CBS activated FXR to regulate BA homeostasis, the FXR antagonist GS was used in rats. Because of abrogation of the FXR after GS treatment, there was a significant serum elevation of ALP, AST, and TBA levels, whereas serum ALT levels were unchanged (Figures 7A,B). In the liver, GS directly decreased protein expression and nuclear translocation of CBS-activated FXR (Figure 7C). Similarly, GS decreased the expression of *Shp* and *Bsep*, classical FXR target genes, and enhanced the expression of *Cyp7a1* in CBS-treated cholestatic rats (Figure 7D). In the intestine, GS decreased protein expression and nuclear translocation of CBS-induced FXR (Figure 7E). *Fgf15*, a direct target gene of FXR in the intestine, was reduced by GS treatment (Figure 7F). Because FGF15 and SHP were both decreased, the reduced expression of *Cyp7a1* by CBS was abrogated by GS administration (Figure 7D). Furthermore, liver histology showed that GS blocked the hepatoprotective effect of CBS, with markedly increased inflammatory cell infiltration, necrosis, and bile duct proliferation observed (Figure 7G). Taken together, these results demonstrated that in rats CBS protected against EE-induced liver injury and dysfunction of BA homeostasis primarily through activating FXR signaling.

## Discussion

**FIGURE 8:**
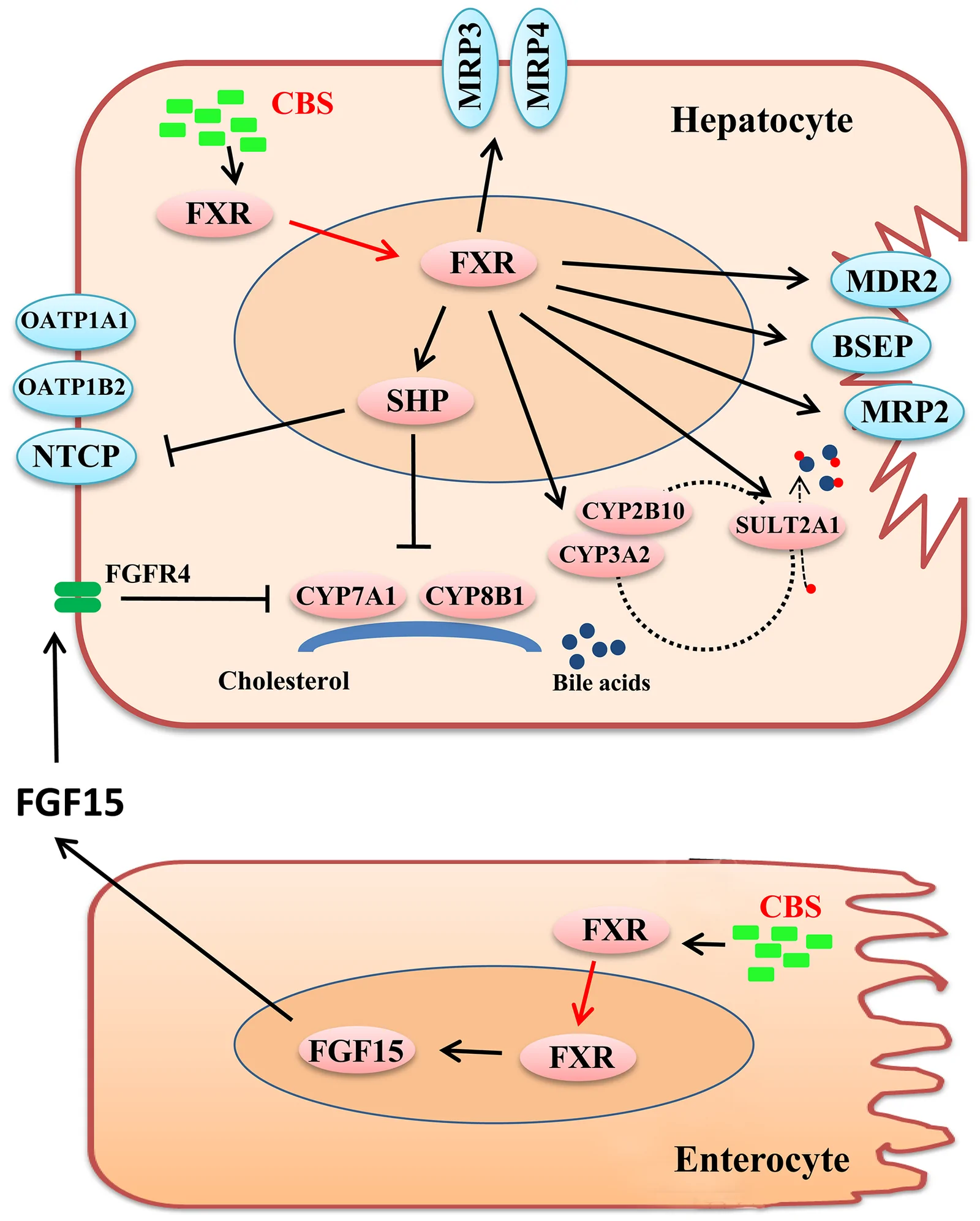
Schematic diagram of potential mechanisms that CBS improves liver injury and hepatic bile acid accumulation in estrogen-induced cholestasis. In liver and intestine, CBS activates protein expression and nuclear translocation of FXR, and then alters the expression of downstream genes. Regarding transporters, CBS decreases NTCP levels to reduce hepatic bile acid (BA) uptake and increases BSEP, MRP2, MRP3, and MRP4 levels to augment BA efflux. Moreover, CBS reduces BA biosynthesis in the liver by repressing BA synthetic enzymes, CYP7A1 and CYP8B1, via hepatic FXR-SHP and intestinal FXR-FGF15 axes. Lastly, CBS increases BA metabolism by increasing the expression of CYP3A2, CYP2B10, and SULT2A1.

Estrogen-induced cholestasis is characterized by impairment of BA uptake and secretion, resulting in the accumulation of toxic BAs and alteration of BA composition, subsequently leading to liver injury (Marrone et al., 2016). In the present study, CBS played a role in protecting against estrogen-induced cholestasis as evidenced by ameliorative liver histology and significant decreases in serum levels of AST, ALT, ALP, and TBA, as well as increases in bile flow and decreases in hepatic BA accumulation. Our data suggested that CBS exhibited hepatoprotective effects and predominantly improved BA homeostasis through FXR-mediated regulation of BA transporters and enzymes (Figure 8). First, CBS decreased hepatic BA uptake and increased BA efflux through downregulation of uptake transporter (NTCP) and upregulation of efflux transporters (BSEP, MRP2, MRP3, and MRP4). In addition, CBS reduced BA biosynthesis in the liver via repressing BA synthetic enzymes (CYP7A1 and CYP8B1) through both hepatic FXR-SHP and intestinal FXR-FGF15 axes. Finally, CBS increased BA metabolism by inducing phase I enzyme (CYP3A2 and CYP2B10) and phase II enzyme (SULT2A1).

Bile acids are not only detergents for lipid absorption, but also signaling molecules, which play essential roles in regulating lipid, glucose, and energy homeostasis (Chiang, 2013). In many livers, the synthesis and clearance of BAs in diseases can be disturbed, thereby potentially leading to alterations in the concentration and composition of BAs in the liver (Liu et al., 2018). The consequential BA accumulation can result in hepatotoxicity and even hepatic necrosis (Woolbright et al., 2015). Therefore, BAs have been considered biomarkers of hepatic diseases and therapeutic efficacy of several drugs (Han et al., 2015; Woolbright et al., 2015; Marrone et al., 2016; Yang et al., 2016; Liu et al., 2018). In this study, the analysis of hepatic BAs showed that major endogenous BAs, such as DCA, TMCA, TCA, and TUDCA, were significantly increased, leading to an abnormal BA composition in EE-induced cholestasis. CBS effectively reversed EE-induced changes in individual BAs and abnormal BA composition in cholestatic rats (Figure 2).

Presently, genomics is widely used in identifying key targets or pivotal pathways for traditional Chinese medicine in treating diseases (Wen et al., 2011). In our study, differentially expressed genes were screened among non-cholestatic, EE, and EE+CBS liver tissues through DNA microarray analysis. We focused on two top enriched pathways, bile secretion, and primary BA synthesis, which are involved in BA homeostasis (Figure 3). In addition, analysis of individual BAs in the liver suggested that anti-cholestatic effects of CBS markedly correlated with mediating BA homeostasis (Figure 2). Therefore, in this study, we focused on the effects of CBS on regulating BA homeostasis, including BA transport, synthesis, and metabolism.

Bile acid transporters and metabolic enzymes play crucial roles in the maintenance of BA homeostasis (Staudinger et al., 2013). BSEP and MRP2 are two main transporters in the canalicular membranes of hepatocytes that are involved in transporting conjugated and unconjugated BAs into bile in human and rodents. This process constitutes the rate-limiting step in hepatic BA excretion (Kast et al., 2002; Plass et al., 2002). Our data showed that EE reduced BSEP and MRP2 protein expression, leading to a decrease in bile flow and hepatic BA accumulation. Moreover, EE decreased the expression of MDR2 to efflux phosphatidylcholine, which is an important ingredient for bile formation (Ghonem et al., 2014). However, CBS administration upregulated the expression of BSEP, MRP2, and MDR2 and recovered the impairment of bile flow and hepatic retention of toxic BAs. In addition, CBS increased the expression of basolateral transporters, MRP3 and MRP4, to increase BAs efflux into the systemic circulation. Hepatic uptake of BAs from the circulation takes place at the basolateral membrane of hepatocytes, and is mediated by NTCP and OATPs (Slijepcevic and van de Graaf, 2017). NTCP takes up most of the reabsorbed BAs in their conjugated form and OATPs mainly uptake some unconjugated BAs into hepatocytes (Slijepcevic and van de Graaf, 2017; Slijepcevic et al., 2018). Combined, previous publications as well as the current study showed that NTCP, OATP1A1, and OTAP1B2 were markedly inhibited by EE to defense against excessive BAs entering hepatocytes (Muchova et al., 2015; Yu et al., 2016), whereas CBS reduced the mRNA and protein expression of NTCP for further inhibition of BA reabsorption. In addition, BA synthetic and metabolic enzymes also play important roles in mediating BA homeostasis. CBS treatment reduced CYP7A1 and CYP8B1 expression leading to suppression of BA synthesis. CBS treatment further increased CYP3A2, CYP2B10, and SULT2A1 expression, which had been shown to contribute to BA detoxification.

In previous studies, it has been shown that several hepatic nuclear receptors, including FXR, PXR, CAR, VDR, LXR, and PPARα participate in regulating BA homeostasis. It has been suggested that PXR and CAR activation results in coordinated stimulation of major hepatic BA metabolizing and detoxifying enzymes (CYP3A, CYP2 isoforms, SULT2A1, CYP7A1) and hepatic key alternative efflux systems (MRP2, MRP3, and MRP4) (Li and Chiang, 2013, 2014). In both mouse and primary human hepatocytes, activation of VDR induced CYP3A and CYP2B expression and repressed CYP7A1 gene expression (Drocourt et al., 2002; Han and Chiang, 2009). LXR, a sterol sensor, affected sensitivity to BA toxicity and cholestasis (Uppal et al., 2007). Cholestatic resistance in LXR transgenic mice was associated with enhancing expression of SULT2A, BSEP, MRP4, and repressing CYP7B1 expression (Uppal et al., 2007). Activation of PPARα had a beneficial effect on cholestatic liver diseases, and was mainly involved in the inhibition of CYP7A1 and upregulation of CYP3A4, UGT1A, and SULT2A1, and induction of MDR2 to increase biliary phospholipids secretion (Ghonem et al., 2015). In previous studies, it was shown that FXR is an extremely important upstream nuclear receptor in the regulation of BA signaling (Massafra et al., 2018). In the liver, FXR directly activated BSEP and SHP, whereas SHP was determined as the upstream gene of NTCP, CYP7A1, and CYP8B1, which can be suppressed by activating FXR–SHP axis (Li and Chiang, 2014). In addition, activation of FXR enhanced CYP3A, CYP2B as well as SULT2A1, BAL, and BAAT expression to increase BA metabolism (Li and Chiang, 2013). In the intestine, FXR activates FGF15, which encodes a hormone that travels to the liver where it interacts with its receptor fibroblast growth factor receptor 4 (Fgfr4), and activates the ERK or JNK signaling cascade to decrease Cyp7a1 and Cyp8b1 expression and inhibit BA synthesis (Zhou et al., 2016). Above all, BA transporters, synthetic and metabolic enzymes may be synergetically controlled by these nuclear receptors.

In the present study, we demonstrated that mRNA and protein levels of hepatic nuclear receptors, including PXR, CAR, VDR, LXR, and PPARα were not significantly different between cholestatic rats or CBS-treated cholestatic rats (Figure 6). In contrast, although the FXR mRNA level was similar between EE and CBS-treated cholestatic rats, FXR protein levels were markedly different in CBS-treated cholestatic rats. CBS administration in cholestatic rats significantly increased FXR protein levels and their nuclear translocation in liver and intestine, and then enhanced the expression of downstream genes (Figure 6). These studies suggest that CBS alleviated EE-induced cholestasis through activating FXR in a posttranscriptional regulation manner. Furthermore, we used FXR antagonist GS to confirm if CBS activated FXR that contributed to recovering BA homeostasis. As expected, GS blocked CBS-induced FXR upregulation and nuclear translocation in liver and intestine, and consequently blocked FXR direct target genes, *Shp* and *Fgf15*. In addition, the beneficial changes in hepatic BA transporters and enzymes, as well as ameliorative serum biomarkers, and liver histology in CBS-treated rats were abrogated by GS (Figure 7).

Farnesoid X receptor is a ligand-activated nuclear receptor that can be activated by free and conjugated-BAs (Ding et al., 2015). The most efficacious BA ligand of FXR is CDCA, followed by LCA, DCA, and CA (Ding et al., 2015). The main constituents of CBS as published in the 2015 edition of the Chinese Pharmacopoeia are BAs and bilirubin. In our previous study, we successfully identified twelve main BAs including CDCA, DCA, and CA as well as their glycine-conjugated and taurine-conjugated derivatives in CBS (Feng et al., 2015). These BAs may be partial bioactive constituents for CBS activating FXR to treat cholestasis. With in-depth research, targeting FXR signaling has been considered to have potential for cholestatic diseases (Fiorucci et al., 2014). In recent years, many FXR agonists have been studied in animal experiments and clinical trials (Fiorucci et al., 2012; Sepe et al., 2015), among which obeticholic acid (OCA) has been successfully approved by U S Food and Drug Administration (FDA) to treat patients with PBC (Kowdley et al., 2018). Thus, there is a great prospect for the development of CBS as an anti-cholestatic drug.

CBS is composed of multiple ingredients, including at least 26 types of BAs (Kai et al., 2018). Future studies are needed to identify the effective substances in CBS and the role of these ingredients on activating FXR in cholestatic animals. Except for dysfunction in BA homeostasis, estrogen cholestasis-induced inflammation, and oxidative stress *in vivo* are also important factors to promote tissue injury (Ozler et al., 2014). Our unpublished studies indicated that CBS markedly alleviated hepatic inflammation and oxidative stress in estrogen-induced cholestatic animals. In this study, even though the effects of CBS on regulating BA homeostasis were suggested, other pathways analyzed by DNA microarray may also play important roles in alleviating cholestasis. Thus, the anti-cholestatic role of CBS needs further investigation, and we will continue to focus on this matter in our future studies.

In summary, CBS improved BA homeostasis in EE-induced cholestatic rats through activating hepatic and intestinal FXR signaling pathways to up-regulate hepatic efflux and metabolism of BAs, and decreased hepatic uptake and synthesis of BAs. Our data suggested that CBS may have considerable potential as a therapeutic in cholestatic liver diseases.

## Author Contributions

DX designated the study, collected and analyzed the data, and wrote the manuscript. JY and YL contributed to data collection. SZ and WH partly performed the experiments. XL analyzed the data. CZ and DL supervised the study. All authors reviewed and approved the manuscript.

## Conflict of Interest Statement

The authors declare that the research was conducted in the absence of any commercial or financial relationships that could be construed as a potential conflict of interest.

## Footnotes

1
https://david.ncifcrf.gov
