# Correction: *Calculus Bovis Sativus* improves bile acid homeostasis via farnesoid X receptor-mediated signaling in rats with estrogen-induced cholestasis

**DOI:** 10.3389/fphar.2026.1918301

**Published:** 2026-08-06

**Authors:** Dong Xiang, Jinyu Yang, Yanan Liu, Wenxi He, Si Zhang, Xiping Li, Chengliang Zhang, Dong Liu

**Affiliations:** Department of Pharmacy, Tongji Hospital Affiliated, Tongji Medical College, Huazhong University of Science and Technology, Wuhan, China

**Keywords:** *Calculus Bovis Sativus*, cholestasis, FXR, 17α-ethinylestradiol, bile acid

Author [Chengliang Zhang] was erroneously spelled as [Chenliang Zhang].

There was a mistake in figure/table [Figure 6F] as published. The β-actin loading control bands in Figure 6F and Figure 7E appear duplicated. Upon review, the original Western blot image from a single exposure contains the β-actin bands for both samples, arranged one above the other: the upper band originally belonged to Figure 6F, and the lower band to Figure 7E. However, during image cropping and figure assembly, the lower band (originally from Figure 7E) was mistakenly reused to serve as the β-actin loading control for Figure 6F as well. As a result, the band displayed in Figure 6F is identical to that in Figure 7E, rather than the intended upper band.

**FIGURE 6:**
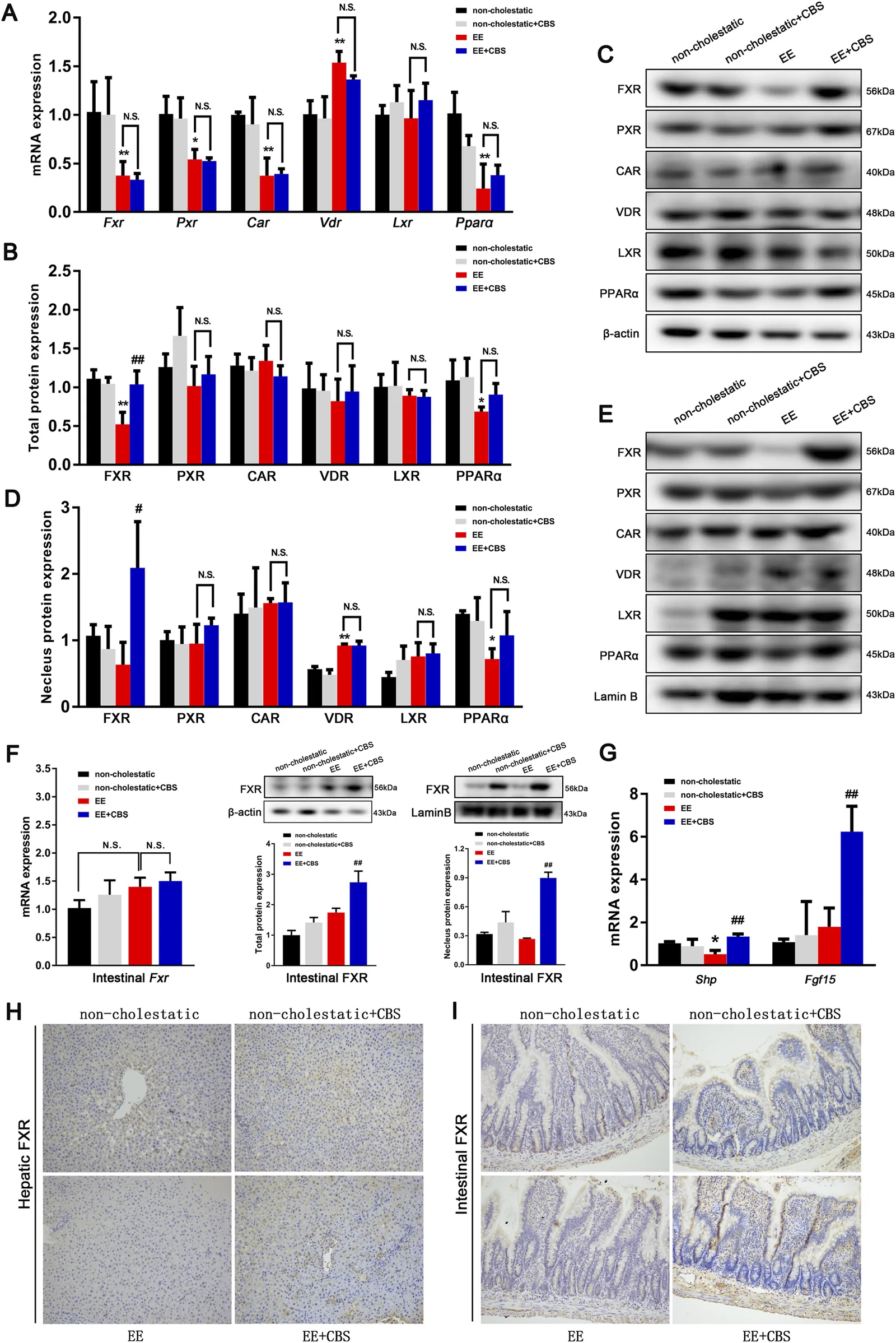
CBS activates the protein expression and nuclear translocation of FXR in liver and intestine. **(A)** mRNA expression of hepatic nuclear receptors Fxr, Pxr, Car, Vdr, Lxr, and Pparα was determined by real-time PCR and normalized to β-actin. **(B,C)** Total protein levels and **(D,E)** nuclear protein levels of hepatic *FXR*, *PXR*, *CAR*, *VDR*, *LXR*, and *PPARα* were determined by Western blot analysis and normalized to β-actin and Lamin B. Representative immunoblot images are shown. **(F)** mRNA, total protein and nuclear protein levels of intestinal FXR were determined using real-time PCR and Western blot analysis and normalized to β-actin and Lamin B. **(G)** mRNA levels of *Shp* in liver and *Fgf15* in intestine were evaluated by real-time PCR and normalized to β-actin. Representative images of immunohistochemical staining of **(H)** hepatic FXR and **(I)** intestinal FXR. Data are presented as the mean ± SD (n = 6). Significant differences compared with the non-cholestatic group, *p < 0.05; **p < 0.01; compared with the 17α-ethinylestradiol (EE) group, #*p* < 0.05; ##*p* < 0.01. N.S., no significance.

It is important to clarify that all quantitative analyses in the original article were performed using the correct bands (the upper band for Figure 6F and the lower band for Figure 7E) based on their grayscale values. Therefore, this figure assembly error has no impact on the statistical results or data accuracy, and does not alter the scientific conclusions of the study. We will submit a corrected Figure 6F and sincerely apologize for this oversight. The corrected figure/table [Figure 6] appears below.

The original version of this article has been updated.

