# Supplementary material for: Calculus Bovis Sativus Improves Bile Acid Homeostasis via Farnesoid X Receptor-Mediated Signaling in Rats With Estrogen-Induced Cholestasis
Source: Front Pharmacol. 2019 Feb 1;10:48. doi: 10.3389/fphar.2019.00048 (PMC6367682; doi:10.3389/fphar.2019.00048)
Supplement: Supplementary file 1 [file Data_Sheet_1.pdf]

Supplementary Material

Calculus Bovis Sativus Improves Bile Acid Homeostasis via Farnesoid X Receptor-Mediated Signaling in Rats with Estrogen-Induced Cholestasis

Dong Xiang[1], Jinyu Yang[1], Yanan Liu[1], Wenxi He[1], Si Zhang[1], Xiping Li[1], Chenliang Zhang[1]*, Dong Liu[1]*

*** Correspondence:**

Dong Liu, MD.

E-mail address: ld2069@outlook.com

Chenliang Zhang, MD.

E-mail address: clzhang@tjh.tjmu.edu.cn

1 Supplementary Figures and Tables

1.1 Supplementary Figures

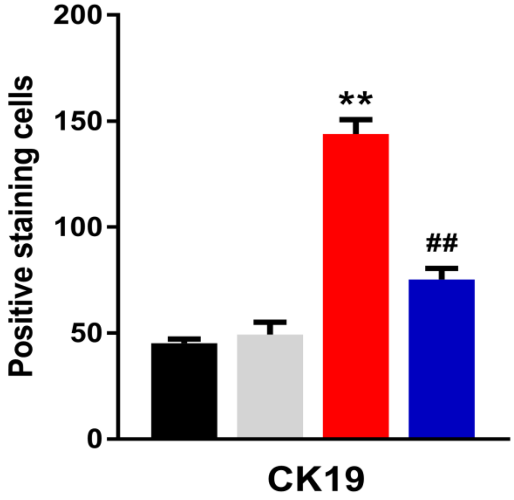

Supplementary Figure 1. Immunohistochemical positive staining cells of CK19 in non-cholestatic or cholestatic rats after vehicle or CBS administration. Positive staining cells were quantified by counting three different fields in each section. Data are presented as the mean ±SD (n=6). Significant differences compared with non-cholestatic group, **$p<0.01$, compared to 17α-ethinylestradiol (EE) group, ##$p<0.01$.

A

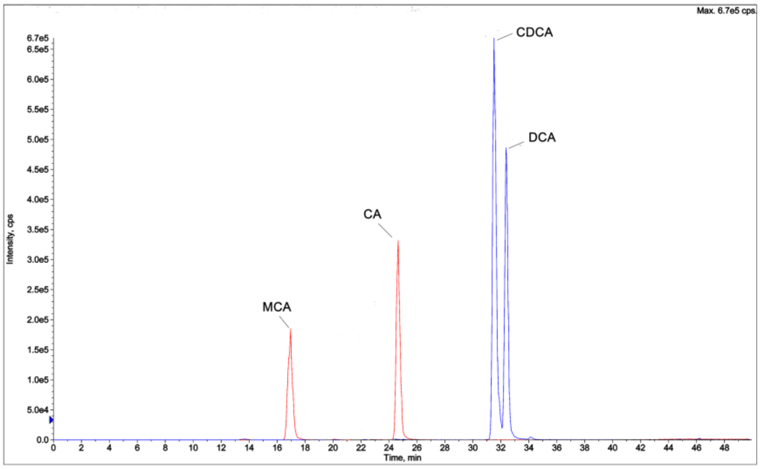

B

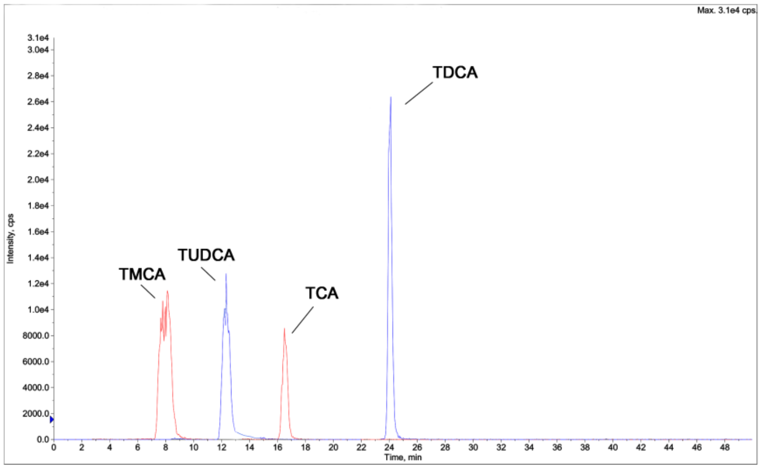

Supplementary Figure 2. Representative LC-MS/MS chromatogram of bile acids in liver. (A) Unconjugated bile acids and (B) conjugated bile acids. Abbreviations: MCA, muricholic acid; CA, cholic acid; CDCA, chenodeoxycholic acid; DCA, deoxycholic acid; TMCA, tauromuricholic acid; TCA, taurocholic acid; TDCA, taurodeoxycholic acid; TUDCA, tauroursodeoxycholic acid.

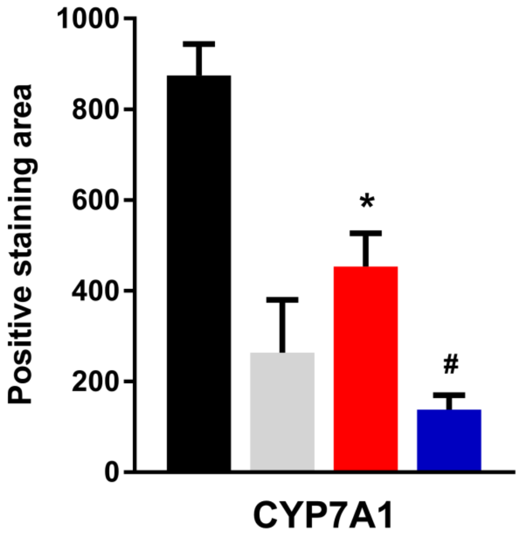

Supplementary Figure 3. Immunohistochemical positive staining area of hepatic CYP7A1 in non-cholestatic or cholestatic rats after vehicle or CBS administration. Positive staining was quantified by counting three different fields in each section. Data are presented as the mean±SD (n=6). Significant differences compared with non-cholestatic group, *p<0.05, compared to 17α-ethinylestradiol (EE) group, #p<0.05.

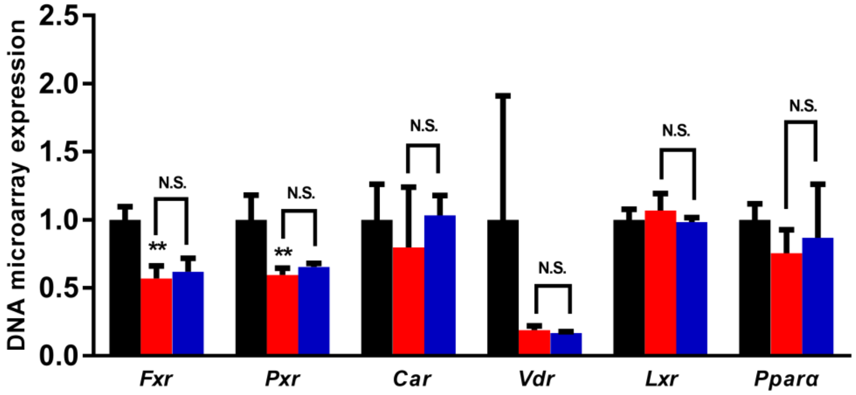

Supplementary Figure 4. Gene expressions of hepatic nuclear receptors Fxr, Pxr, Car, Vdr, Lxr and Pparα analyzed by DNA microarray. Data are presented as the mean±SD (n=3). Significant differences compared with non-cholestatic group, **p<0.01.

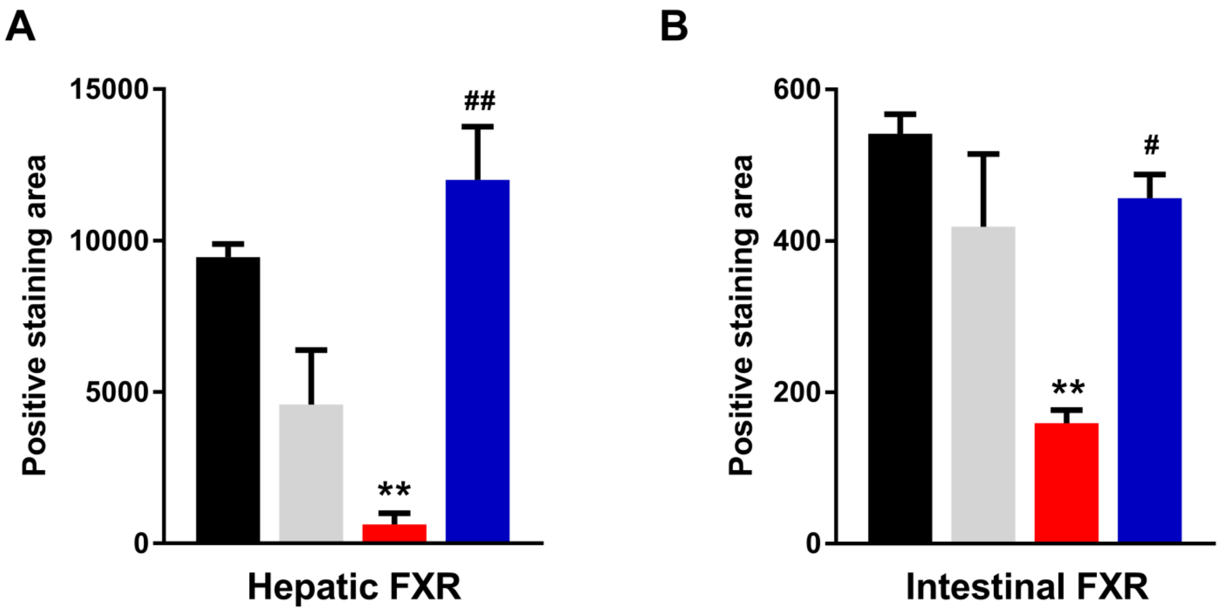

Supplementary Figure 5. Immunohistochemical positive staining area of hepatic FXR and intestinal FXR in non-cholestatic or cholestatic rats after vehicle or CBS administration. Positive staining was quantified by counting three different fields in each section. Data are presented as the mean±SD (n=3). Significant differences compared with non-cholestatic group, **p<0.01, compared to 17α-ethinylestradiol (EE) group, #p<0.05; ##p<0.01.

1.2 Supplementary tables

Supplementary Table 1 The primer sequences used for real-time PCR assay in rats

Gene	Accession NO.	Protein	Forward primer (5’ →3’)	Reverse primer (5’ →3’)	Product length
Slc10a1	NM_017047.1	NTCP	TGCACCATAGGGATCGTCCTC	GATGCTGTTGCCCACATTGA	135
Slco1a1	NM_017111.1	OATP1A1	TGGGGAAGGTTGCTGGCCCAATTT	GGTGGTTAATCCAGCAACTGCTGC	659
Abcc3	NM_080581.1	MRP3	TCCCACTTCTCGGAGACAGTAAC	CTTAGCATCACTGAGGACCTTGAA	87
Abcc4	NM_133411.1	MRP4	GAAGGAAAATGAGGAAGCAGAG	GGATGACTGTTGAGACCAAATC	100
Slco1b2	NM_001270587.1	OATP1B2	AGACGTTCCCATCACAACCAC	GCCTCTGCAGCTTTCCTTGA	68
Abcc2	NM_012833.2	MRP2	CTCGGTCTTATGCGGCGTATTC	CCGTGACTGATGGAGTTTGTGTT	179
Abcb11	NM_031760.1	BSEP	CGTGCTTGTGGAAGAAGTTG	GGGAGTAGATGGGTGTGACTG	131
Abcb4	NM_012690.2	MDR2	CTGTAGCGGGAATTGTTG	GATGCCGTAGATGTGAGC	215
Cyp7a1	NM_012942.2	CYP7A1	GTCCGGATATTCAAGGATGC	GGGAATGCCATTTACTTGGA	107
Cyp8b1	NM_031241.1	CYP8B1	ATGAAGGCTGTGCGAGAG	TCTCTTCCATCACGCTGTC	127
Cyp3a2	NM_153312.2	CYP3A2	TGACTGCTCTTGATGCATGGTT	ATCACAGACCTTGCCAACTCCT	104
Cyp2b10	XM_017594657.1	CYP2B10	GGTGGAGGAACTGCGGAAAT	TGATGCACTGGAAGAGGAAGGT	65
Sult2a1	NM_131903.1	SULT2A1	AGGAACGAACTGGCTGATTG	ATGGGAAGATGGGAGGTCAT	171
Slc27a5	NM_024143.2	BAL	TGCCCTTGCTACACTCTG	TGGTCCCTGAAGTATAGATG	264
Baat	NM_017300.2	BAAT	CTGTCGAACTACGGTTTTGGCGAA	TCAGGCCTGTGACCCGGATA	136
Nr1h4	NM_021745.1	FXR	TGAGCGTCTACAGCGAAAGTG	GGGATGGTGGTCTTCAAATAAG	121
Nr1i3	XM_017598932.1	CAR	ACCAGTTTGTGCAGTTCAGG	CTTGAGAAGGGAGATCTGGT	197
Nr1i2	NM_052980.2	PXR	GACGGCAGCATCTGGAACTAC	TGATGACGCCCTTGAACATG	112
Nr1i1	NM_017058.1	VDR	GCCCCTCATAAAGTTCCAGGTG	GGATAGGCGGTCCTGAATGG	148
Nr1h3	XM_006234534.2	LXR	TCAGCATCTTCTCTGCAGACCGG	TCATTAGCATCCGTGGGAACA	144
Pparα	XM_017594681.1	PPARA	CGGGTCATACTCGCAGGAAAG	TGGCAGCAGTGGAAGAATCG	155
Nr0b2	NM_057133.1	SHP	CTCGGTTTGCATACAGTGTTTGAC	GCATATTGGCCTGGAGGTTTT	75
Fgf15	NM_130753	FGF15	ACGGGCTGATTCGCTACTC	TGTAGCCCAAACAGTCCATTTCCT	66

Supplementary Table 2 linear regression, correlation coefficient, and linearity range of the bile acid determination

Bile acids	Linear regression equation($1/x^2$)	Correlation coefficient	linearity range （μmol/L）
TCA	y=0.548x-0.0108	r=0.9951	0.275-100.0
DCA	y=6.6x+0.471	r=0.9972	0.275-100.0
TDCA	y=0.707x-0.0131	r=0.9978	0.275-100.0
T-MCA	y=0.57x-0.00806	r=0.9954	0.275-100.0
MCA	y=8.25x-0.00303	r=0.9986	0.0069-10.00
CA	y=7.96x+0.000283	r=0.9974	0.0069-10.00
CDCA	y=5.92x-0.00455	r=0.9982	0.0069-10.00
TUDCA	y=0.714x+0.000566	r=0.9974	0.0069-10.00

Abbreviations: MCA, muricholic acid; CA, cholic acid; CDCA, chenodeoxycholic acid; DCA, deoxycholic acid; TMCA, tauromuricholic acid; TCA, taurocholic acid; TDCA, taurodeoxycholic acid; TUDCA, tauroursodeoxycholic acid.

Supplementary Table 3 Summary of intra-day accuracy, inter-day accuracy, precision, and recovery of the bile acid determination

Bile acid	Quality control	Concentration (μmol/L)	Intra-day(n=5)		Inter-day(n=5)		Recovery (%)
			Accuracy (%)	RSD(%)	Accuracy (%)	RSD(%)	
TCA	LQC	11.1	102.6	8.6	98.4	4.3	89.6
	MQC	50.0	90.3	4.3	91.4	3.1	87.4
	HQC	80.0	96.5	9.1	94.3	5.9	97.5
DCA	LQC	11.1	103.6	6.3	97.5	5.8	91.1
	MQC	50.0	94.2	2.1	96.3	2.4	95.3
	HQC	80.0	89.3	8.7	91.5	6.3	94.2
TDCA	LQC	11.1	98.4	11.6	94.2	5.6	100.3
	MQC	50.0	101.2	8.9	99.4	4.9	96.4
	HQC	80.0	91.1	3.7	93.5	8.5	97.6
TMCA	LQC	11.1	93.6	9.4	97.1	2.9	91.2
	MQC	50.0	98.1	13.4	97.2	7.4	97.3
	HQC	80.0	91.7	11.3	89.4	4.8	92.7
MCA	LQC	1.11	89.5	9.4	93.5	10.6	89.6
	MQC	5.00	97.3	5.6	95.4	6.7	91.9
	HQC	8.00	96.2	7.3	94.7	4.1	98.2
CA	LQC	1.11	95.4	4.0	97.1	5.4	90.4
	MQC	5.00	97.2	8.3	95.0	5.3	93.3
	HQC	8.00	91.3	12.6	90.9	8.9	88.7
CDCA	LQC	1.11	94.1	2.9	97.2	7.4	95.7
	MQC	5.00	99.3	8.9	98.5	8.1	92.1
	HQC	8.00	100.1	4.3	102.5	5.4	90.6
TUDCA	LQC	1.11	92.4	5.9	89.1	8.9	98.9
	MQC	5.00	94.8	8.3	91.7	7.3	100.8
	HQC	8.00	88.3	7.8	92.6	4.4	101.6

Abbreviations: LQC, low quality control; MQC, middle quality control; HQC, high quality control.

Supplementary Table 4 Gene ontology analysis of differentially expressed genes of non-cholestatic, 17α-ethinylestradiol (EE) and EE+*Calculus Bovis Sativus* (CBS) groups

Category	Term	Count	-lg(PValue)	Genes
BP	GO:0042493~response to drug	10	3.18	ECE1, MVD, NPPC, FZD1, COL1A1, AK4, CALR, AOC1, PFAS, NNMT
BP	GO:1903298~negative regulation of hypoxia-induced intrinsic apoptotic signaling pathway	2	1.74	NOL3, PINK1
BP	GO:0000122~negative regulation of transcription from RNA polymerase II promoter	9	1.69	CEBPA, TSC22D3, THRB, TBX3, HNRNPA2B1, PER1, CALR, CELA1, NFIB
BP	GO:0050821~protein stabilization	4	1.67	ATP1B1, PINK1, CCT6A, CALR
BP	GO:0060662~salivary gland cavitation	2	1.57	TGM2, NFIB
CC	GO:0070062~extracellular exosome	26	3.62	ATP1B1, THRB, POLG, HNRNPA2B1, IGFALS, PDIA6, PPCS, TFG, CCT6A, AK4, CALR, RPS2, PFAS, PTER, ACTG1, APP, ECE1, GSTK1, PTP4A1, TGFBI, TGM2, VNN1, RGD1309534, SCARB1, LAMC1, AOC1
CC	GO:0031012~extracellular matrix	6	2.20	TGFBI, TGM2, COL1A1, CCT6A, LAMC1, CALR
CC	GO:0005790~smooth endoplasmic reticulum	3	2.11	APP, PDIA6, CALR
CC	GO:0005925~focal adhesion	7	2.01	ACTG1, FZD1, TGM2, ZYX, CALR, PALLD, RPS2
CC	GO:0005794~Golgi apparatus	10	1.87	SLC39A14, APP, BLZF1, ING2, CABP2, SGPP1, COL1A1, CALR, TRIP11, CALU
MF	GO:0005515~protein binding	17	2.66	CEBPA, NOL3, MAFB, HNRNPA2B1, FZD1, CCNL1, PINK1, PDIA6, CCT6A, CALR, APP, BLZF1, CAPN10, TGM2, PER1, COL1A1, ERRFI1
MF	GO:0019900~kinase binding	4	2.06	CEBPA, NOL3, PER1, ERRFI1
MF	GO:0042803~protein homodimerization activity	10	1.93	CEBPA, CHKA, ECE1, MASP1, THRB, MVD, NPPC, FZD1, SCARB1, AOC1
MF	GO:0005506~iron ion binding	5	1.84	CYP2B2, CYP2B15, CALR, CYP8B1, PHF8
MF	GO:0004305~ethanolamine kinase activity	2	1.74	CHKA, ETNK1

Abbreviations: BP-biological process; CC-cellular component; MF-molecular function.

Supplementary Table 5 Pathways analysis of differentially expressed genes of non-cholestatic, 17α-ethinylestradiol (EE) and EE+*Calculus Bovis Sativus* (CBS) groups.

Term	Count	-lg(PValue)	Genes
Metabolic pathways	15	2.323349	CHKA, CYP2B2, HYKK, MVD, POLG, NDUFA6, PPCS, ETNK1, CYP2B15, AK4, CYP8B1, AOC1, PFAS, SLC27A5, NNMT
Bile secretion	4	2.217689	ATP1B1, ABCC3, SCARB1, SLC27A5
Primary bile acid biosynthesis	2	1.090755	CYP8B1, SLC27A5
Chemical carcinogenesis	3	1.08231	CYP2B2, GSTK1, CYP2B15
Phagosome	4	1.077844	ACTG1, SCARB1, CALR, CD209B
Pantothenate and CoA biosynthesis	2	1.019434	VNN1, PPCS
Focal adhesion	4	1.009053	ACTG1, COL1A1, ZYX, LAMC1
